# Stillbirth rate and associated factors at the Bamenda Regional hospital, North-West region, Cameroon, from 2018 to 2022: a case control study

**DOI:** 10.1186/s12884-024-06486-z

**Published:** 2024-04-12

**Authors:** Achuo Ascensius Ambe Mforteh, Dobgima Walter Pisoh, Merlin Boten, Nkomodio Enanga-Linda Andoh, Theodore Yangsi Tameh, Audrey-Fidelia Eyere Mbi-Kobenge, Kingsley Sama Ombaku, William Ako Takang, Robinson Enow Mbu

**Affiliations:** 1 Faculty of Health Sciences, University of Bamenda, P.O. Box 39, Bamenda, Cameroon; 2 Bamenda Regional Hospital, Bamenda, Cameroon; 3 Faculty of Medicine and Biomedical Sciences, University of Yaoundé 1, Yaounde, Cameroon; 4 Yaoundé Gyneco-Obstetric and Paediatric Hospital, Yaounde, Cameroon

**Keywords:** Stillbirth rate, Risk factors, Stillbirth, Case–control, Bamenda, Cameroon

## Abstract

**Background:**

Stillbirth is a common adverse pregnancy outcome worldwide, with an estimated 2.6 million stillbirths yearly. In Cameroon, the reported rate in 2015 was 19.6 per 1000 live births. Several risk factors have been described, but region-specific risk factors are not known in the northwest region of Cameroon. This study aims to determine the stillbirth rate and associated factors at the Bamenda Regional hospital, North-West region of Cameroon.

**Materials and methods:**

A Hospital-based case‒control study conducted from December 2022 to June 2023 on medical files from 2018 to 2022 at the Bamenda Regional Hospital. Cases were women with stillbirths that occurred at a gestational age of ≥ 28 weeks, while controls were women with livebirths matched in a 1:2 (1 case for 2 controls) ratio using maternal age. Sociodemographic, obstetric, medical, and neonatal factors were used as exposure variables. Multivariable logistic regression was used to determine adjusted odds ratios of exposure variables with 95% confidence intervals and a p value of < 0.05.

**Results:**

A total of 12,980 births including 116 stillbirths giving a stillbirth rate of 8.9 per 1000 live births. A hundred cases and 200 controls were included. Factors associated with stillbirths after multivariable analysis include nulliparity (aOR = 3.89; 95% CI: 1.19–12.71; *p* = 0.025), not attending antenatal care (aOR = 104; 95% CI: 3.17–3472; *p* = 0.009), history of stillbirth (aOR = 44; 95% CI: 7-270; *p* < 0.0001), placenta abruption (aOR = 14; 95% CI: 2.4–84; *p* = 0.003), hypertensive disorder in pregnancy (aOR = 18; 95% CI: 3.4–98; *p* = 0.001), malaria (aOR = 8; 95% CI: 1.51-42; *p* = 0.015), alcohol consumption (aOR = 9; 95% CI: 1.72-50; *p* = 0.01), birth weight less than 2500 g (aOR = 16; 95% CI: 3.0–89; *p* = 0.001), and congenital malformations (aOR = 12.6; 95% CI: 1.06–149.7;*p* = 0.045).

**Conclusion:**

The stillbirth rate in BRH is 8.9 per 1000 live births. Associated factors for stillbirth include nulliparity, not attending antenatal care, history of stillbirth, placental abruption, hypertensive disorder in pregnancy, malaria, alcohol consumption, birth weight less than 2500 g, and congenital malformations. Close antenatal care follow-up of women with such associated factors is recommended.

## Background

In low- and middle-income countries (LMICs), stillbirth is defined as death of a baby when birth weight ≥ 1000 g or gestational age ≥ 28 completed weeks or body length ≥ 35 cm before or during birth [1]. Although the International Classification of Diseases of the World Health Organization (WHO) uses birth weight ≥ 500 g, or gestational age ≥ 22 completed weeks, or body length ≥ 25 cm, the former definition is used for international comparison [1, 2].

Globally, there are an estimated 2.6 million stillbirths each year, with the vast majority (98%) occurring in low- and middle-income countries [3]. In Africa, the stillbirth rate is estimated at 24 per 1000 live births [4], with sub-Saharan Africa having the highest rate, estimated at 28.7 per 1000 live births [5]. A study performed in neighbouring Nigeria estimated the stillbirth rate at 38 per 1000 live births in 2012 and 27 per 1000 live births in 2013 [6]. The rates in Cameroon were reported to be 20 per 1000 live births in 2004, 25.6 per 1000 live births in 2009 and 19.6 per 1000 live births in 2015 [7]. In a hospital-based study on stillbirth, carried out in the Buea and Limbe regional hospitals in the South West Region of Cameroon in 2020, the estimated rates of stillbirths in both hospitals were 34 and 36 per 1000 livebirths, respectively [8]. These values are high compared to the target set by the WHO’s Every New-Born Action Plan in all countries by 2030, which stands at ≤ 12 per 1000 live births, and the current WHO estimates for Cameroon [7]. In addition to this adverse perinatal outcome of stillbirth, maternal adverse outcomes such as anxiety, depression, posttraumatic stress disorder, and stigmatization have been reported [9].

Several risk factors for stillbirth have been identified and include older maternal age (> 35 years), obesity, smoking, obstructed labour, intrauterine growth restriction, diabetes, hypertension and maternal infections [10]. Nearly 60% of stillbirths occur in rural families who generally have limited access to medical care [3]. More than half of all stillbirths occur during labour, and the majority of such stillbirths could be prevented by the provision of adequate maternal healthcare [3].

Several strategies to reduce the rate of stillbirth have been implemented. Such strategies include the use of sulfadoxine and pyrimethamine as intermittent preventive treatment against malaria in endemic areas, detecting and treating syphilis, nutritional supplementation, and increasing access to emergency obstetric care [11]. However, enough competent birth attendants and resources for facility deliveries are not available in many places, leading to the proposal of educating community birth attendants to offer basic care and assessing the need for referral as an intermediate solution [11]. Better treatment of medical conditions such as diabetes and hypertension has resulted in a significant reduction in stillbirths in high-income countries, and if effective treatment of these and other medical causes of stillbirth are well practiced, similar results may be achieved in LMICs countries [11]. Other risk factors must also be considered in implementing an effective strategy to reduce stillbirth. No such study on the rate and risk factors for stillbirth has been conducted in the northwest region of Cameroon. This study aims to determine the stillbirth rate and associated factors at the Bamenda Regional hospital, North-West region of Cameroon.

## Methods

### Study design, period and setting

This was a 1:2 hospital-based matched case‒control study with cases and controls selected from 1st January 2018 to 31st December 2022. The study was conducted from December 2022 to June 2023 in the Obstetrics and Gynaecology Department of the Bamenda Regional Hospital (BRH), a second level referral hospital. The BRH is situated in Bamenda, the capital city of the North-West region of Cameroon. The BRH serves as a teaching hospital for the Faculty of Health Sciences, University of Bamenda, and the main referral hospital of the region. It is a public hospital with a relatively lower fee for services compared to non-public health facilities which probably makes it more solicited by clients than other hospital. In addition, it is the only facility with a constant effective presence of gynaecologists and obstetricians and midwives in the region. The staff strength of the Obstetrics and Gynecology Department includes 3 obstetricians-gynaecologists, 2 general practitioners, 15 midwives, and 12 state registered nurses. The Departmment is made up of various services, including labour and delivery, postnatal care, an antenatal clinic, outpatient consultation, family planning, and an inpatient service. The labour and delivery service has a cardiotocograph for monitoring at-risk parturients. The BRH also has a neonatology unit manned by three paediatricians and 11 state registered nurses. In addition to being the main referral hospital and having a high patient flow, this hospital was chosen due to the availability of files during the study period.

### Study population

**Fig. 1:**
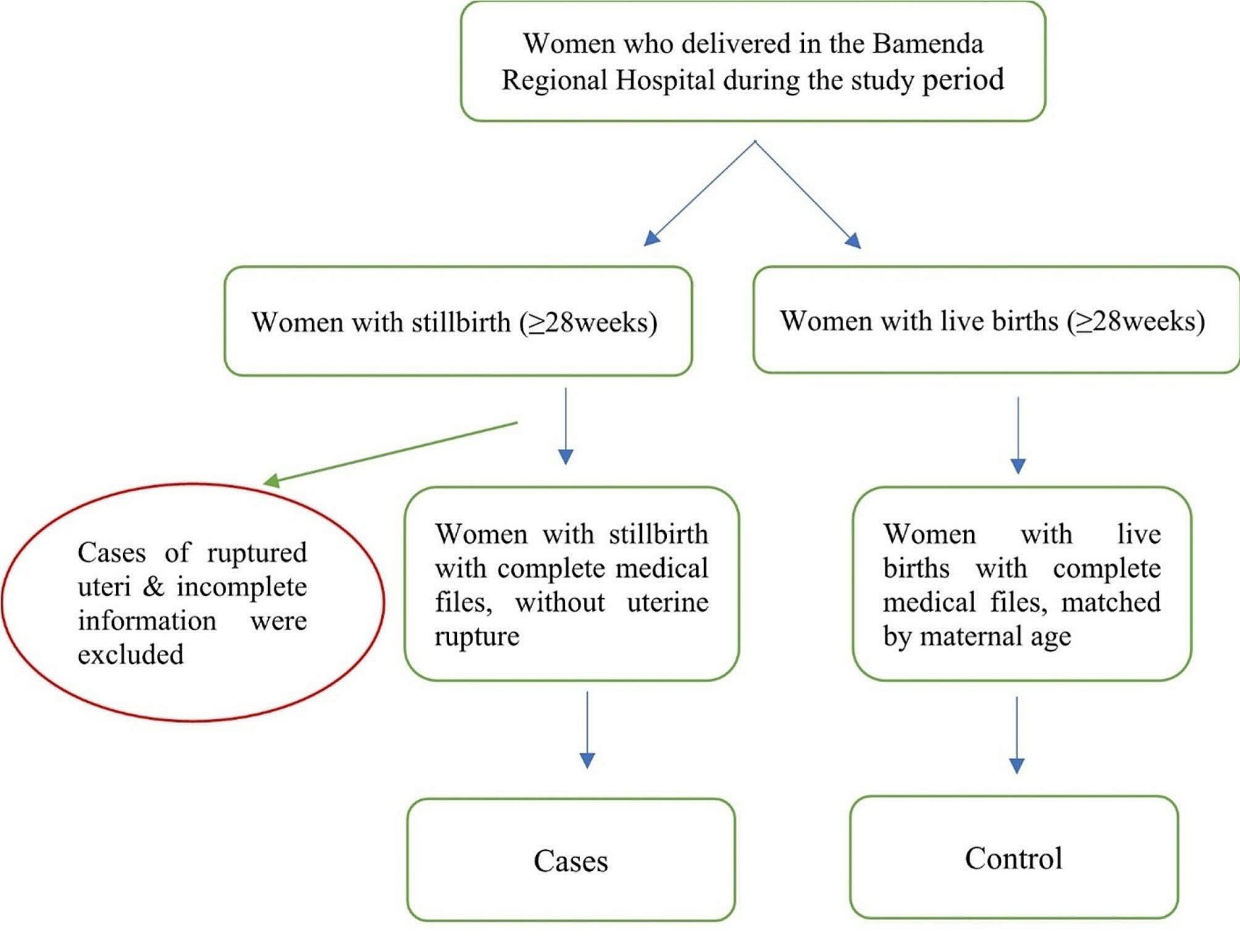
Flow diagram for study population

Our target population consisted of pregnant women in Bamenda, while our parent population from which our study population was obtained consisted of pregnant women who delivered in the BRH. Participants in the study were recruited retrospectively using a delivery register for the study period. For cases, the inclusion criteria were stillbirth and gestational age ≥ 28 weeks while the exclusion criteria were incomplete files and those for whom the reason for stillbirth was uterine rupture. For controls, inclusion criteria were women with live births on the day of delivery or within seven days preceding the stillbirth, matched by maternal age and with complete files. Matching was done using the age range of 15–19, 20–24, 25–29, 30–34, 35–39, and greater than or equal to 40 years. For each case, two controls were selected. Where there were more than two controls eligible, two controls with the smallest age difference between control and case were selected and the others excluded. Figure 1 below illustrates the study population selection.

### Sampling and sample size estimation

A minimum sample size of 88 cases was assumed from a similar study by Suleiman et al. [12]. All files of cases were sorted out and those that met the eligibility criteria were retained. Corresponding controls were sorted out using delivery registers to identify the controls.

### Data collection and study variables

A preestablished data collection form was used to collect sociodemographic, obstetrical, medical, and neonatal data from birth registers and medical files. The outcome variable was birth outcome categorized into stillbirth and live birth. The exposure variables consisted of marital status, occupation, religion, gravidity, parity, interpregnancy period, previous history of stillbirth, attendance of antenatal care (ANC), number of antenatal care visits, gestational age at first ANC, gestational age of stillbirth, ultrasound during pregnancy, TORCH serology (toxoplasmosis, rubella, cytomegalovirus, herpes simplex) during pregnancy, hypertensive disorder in pregnancy, diabetes, medical complications during pregnancy, intermittent preventive treatment (IPT), smoking, alcohol consumption, baby’s birth weight, sex, and congenital malformation.

### Data management and statistical analysis

Data were entered into Microsoft Office Excel 2016 and exported into the statistical Package for Social Sciences (SPSS) software version 26 for analysis. Continuous variables, including age, gestational age, parity, gravidity, interpregnancy period, number of antenatal care visits, gestational age at booking visit, number of ultrasounds, number of IPTs, gestational age of rupture of membranes, and birth weight, were categorized and used as such for analysis. Missing data were not included in the final analysis. The stillbirth rate was calculated as the number of stillbirths per 1000 live births. The distribution of exposure variables amongst the cases and controls was described using frequency tables. Associations between exposure variables and stillbirth were assessed using the chi-square test or Fisher’s exact test in cases where at least one expected frequency was less than 5. Variables with p values less than 0.1 were subsequently included in a multivariable logistic regression to determine adjusted odds ratios while controlling for confounding factors. Statistical significance was set at *p* < 0.05. Adjusted odds ratios and 95% confidence intervals were reported.

### Ethical considerations

Ethical clearance was obtained from the Institutional Review Board of the University of Bamenda (Reference number: 23,000,038/Uba/D-FHS). Informed consent was not sought but giving the retrospective nature of the study design, this was waived by the University of Bamenda Institutional Review Board. Administrative authorization was obtained from the administration of the Bamenda Regional Hospital (Reference number: R005/MPH/RDPH/RHB/024). Patient confidentiality was assured by using codes to replace names, and no information that could lead to the identification of participants such as contact numbers, was copied on the data entry form.

### Definition of operational terms

Stillbirth: foetal demise occurring at ≥ 28 completed weeks of gestation and before birth.

Live birth: baby delivered at ≥ 28 completed weeks of gestation with signs of live.

## Results

A total of 13,096 deliveries were recorded during the 5-year study period, consisting of 116 stillbirths and 12,980 livebirths, giving a stillbirth rate of 8.9 per 1000 livebirths (95% confidence interval: 7.4–10.7). Of the 116 stillbirths, 16 were excluded due to incomplete information on the key variable of gestational age and stillbirths due to ruptured uterus. Analysis was performed for 100 cases with corresponding 200 controls. Maternal age ranged from 15 to 43 years, with a mean of 28.5 ± 6.5 years. The age group of 25–29 was the most represented, with 32 (32%) cases in this age group. Fifty-five (55%) of the cases were single, 58 (58%) were employed, and 93 (93%) were Christians. For the controls, 61 (30.5%) were single, 133 (66.5%) were employed, and 190 (95%) were Christians. Table 1 below shows the sociodemographic characteristics of the study population.

**Table 1:** Sociodemographic characteristics of the mother

Variables	Category	Frequency	Percentage (%)
Age (years)	15–19	21	7
	20–24	72	24
	25–29	96	32
	30–34	57	19
	35–39	24	8
	≥40	30	10
Marital status	Single	116	38.7
	Married	184	61.3
Occupation	Student	57	19
	Employed	191	63.7
	Unemployed	52	17.3
Religion	Christian	283	94.3
	Muslim	17	5.7

### Sociodemographic determinants of stillbirth

Being single was positively associated with stillbirth compared to being married, with the odds of having a stillbirth being 2.8 times higher for singles compared to being married (OR: 2.79; 95% CI: 1.69–4.57, *p* < 0.001). The secondary level of education was negatively associated with stillbirth (OR: 0.40; 95% CI: 0.20–0.79, *p* = 0.007) (Table 2).

**Table 2:** Sociodemographic factors associated with stillbirth

Variables	Category	Cases n (%)	Controls n (%)	OR [95% CI]	P value
Marital status	Single	55 (55.0)	61 (30.5)	2.79 [1.69–4.57]	**< 0.001**
	Married	45 (45.0)	139 (69.5)	1	
Level of education	No formal	10 (10.2)	6 (3.0)	1.96[0.63–6.63]	0.23
	Basic	25 (25.5)	30 (15.1)	1	
	Secondary	28 (28.6)	85 (42.7)	0.40[0.20–0.79]	**0.007**
	University	35 (35.7)	78 (39.2)	0.54[0.28–1.06]	0.066
Occupation	Student	22 (22.0)	35 (17.5)	1.44 [0.77–2.67]	0.243
	Employed	58 (58.0)	133 (66.5)	1	
	Unemployed	20 (20.0)	32 (16.0)	1.43 [0.75–2.71]	0.268
Religion	Christian	93 (93.0)	190 (95.0)	1	
	Muslim	7 (7.0)	10 (5.0)	1.44 [0.50–3.92]	0.480

OR: Odds ratio, CI: confidence interval

### Obstetrical determinants of stillbirth

Nulliparity (Parity of 0) (OR: 1.90; 95% CI:1.10–3.27, *p* = 0.02), no ANC visit compared to ANC visit at regional hospital (OR:16.93; 95% CI:6.03–57.1, *p* < 0.0001), ANC location in health centre (OR: 1.89; 95% CI:1.01–3.53, *p* = 0.047), ANC location in district hospital (OR:2.41; 95% CI:1.13–5.13, *p* = 0.023), no ANC visits compared to 5 or more ANC visits (OR:13.34; 95% CI:4.96–43.4, *p* < 0.001), history of stillbirth (OR:9.94; 95% CI:4.12–28.1, *p* < 0.001), Zero ultrasound during pregnancy (OR:3.5; 95% CI:1.28–11.4, *p* = 0.013), premature rupture of membranes < 37 weeks (OR:8.4; 95% CI:1.83–38.57, *p* = 0.006), oligohydramnios (OR:4.85; 95% CI:1.45–16.15, *p* = 0.012), not doing the TORCH serology (OR:2.29; 95% CI:1.38–3.88, *p* = 0.001), having an infection during pregnancy (OR:2.98; 95% CI: 1.62–5.52, *p* < 0.001), and placenta abruption (OR:26.1; 95% CI:5.04–640, *p* < 0.001) were significantly associated with stillbirth in the bivariate analysis (Table 3).

**Table 3:** Obstetrical factors associated with stillbirth

Variables	Category	Cases n (%)	Controls n (%)	OR [95% CI]	P value
Parity	0	34 (34.0)	45 (22.5)	1.90 [1.10–3.27]	**0.02**
	1–3	56 (56.0)	141 (70.5)	1	
	>3	10 (10.0)	14 (7.0)	1.79 [0.73–4.30]	0.181
ANC location	No ANC	22 (22)	5 (2.5)	16.93 [6.03–57.1]	**< 0.001**
	Health centre	40 (40.0)	86 (43.0)	1.87 [1.01–3.56]	**0.046**
	District Hospital	19 (19.0)	32 (16.0)	2.39 [1.11–5.16]	**0.021**
	Regional Hospital	19 (19.0)	77 (38.5)	1	
Number of	0	22 (22.0)	5 (2.5)	13.34 [4.96–43.4]	**< 0.001**
ANC done	1–2	9 (9.0)	29 (14.5)	0.99 [0.40–2.27]	*0.965*
	3–4	38 (38.0)	68 (34.0)	1.76 [1.00-3.12]	*0.048*
	≥ 5	31 (31.0)	98 (49.0)	1	
GA at 1st ANC (weeks)*	≤ 12	15 (20.3)	24 (12.4)	1	
	> 12	59 (79.7)	170 (87.6)	0.55 [0.27–1.53]	0.101
History of stillbirth	Yes	24 (24.0)	6 (3.0)	9.94 [4.12–28.1]	**< 0.001**
	No	76 (76.0)	194 (97.0)	1	
Number of Ultrasound done**	0	46 (49.9)	51 (30.9)	3.50 [1.28–11.4]	**0.013**
	1–2	42 (45.2)	94 (57.0)	1.75 [0.65–5.64]	0.271
	≥ 3	5 (5.4)	20 (12.1)	1	
Chorio-	Yes	2 (2.0)	0 (0.0)	∞[U-∞]	0.110^F^
amnionitis	No	98 (98.0)	200 (100.0)	1	
Use of IPT***	Yes	76 (76.0)	168 (84.0)	0.61 [0.31–1.21]	0.156
	No	17 (17.0)	23 (11.5)	1	
Number IPT***	0	17 (18.3)	23 (12.0)	1.49 [0.74–3.01]	0.265
	1	4 (4.3)	17 (8.9)	0.48 [0.15–1.48]	0.198
	2	15 (16.1)	36 (18.8)	0.84 [0.43–1.66]	0.617
	≥ 3	57 (61.3)	115 (60.2)	1	
**PROM**	Yes	17 (17.0)	19 (9.5)	1.95 [0.95–3.96]	0.060
	No	83 (83.0)	181 (90.5)	1	
GA at PROM (weeks)	28 weeks to < 37	12 (70.6)	4 (22.2)	8.40 [1.83–38.57]	**0.006**
	≥ 37	5 (29.4)	14 (77.8)	1	
Oligo-hydramnios	Yes	9 (9.0)	4 (2.0)	4.85 [1.45–16.15]	**0.012**
	No	91 (91.0)	196 (98. 0)	1	
Post term pregnancy	Yes	9 (9.0)	15 (7.5)	1.22 [0.51–2.89]	0.652
	No	91 (91.0)	185 (92.5)	1	
TORCH Serology	Not done	71 (71.0)	103 (51.5)	2.29 [1.38–3.88]	**0.001**
	Done	29 (29.0)	97 (48.5)	1	
Infection in pregnancy	Yes	29 (29.0)	24 (12.0)	2.98 [1.62–5.52]	**< 0.001**
	No	71 (71.0)	176 (88.0)	1	
Placenta Previa	Yes	4 (4.0)	4 (2.0)	2.04 [0.45–9.21]	0.447
	No	96 (96.0)	196 (98.0)	1	
Placenta abruption	Yes	13 (13.0)	1 (0.5)	26.1 [5.04–640.5]	**< 0.001**
	No	87 (87.0)	199 (99.5)	1	

ANC: antenatal care; CI: Confidence interval; GA: Gestational age; IPT: intermittent preventive treatment for malaria using sulfadoxine-pyrimethamine; OR: Odds ratio; PROM: Premature rupture of membranes; TORCH: Toxoplasmosis, rubella, cytomegalo virus, herpes

*Information on GA at 1st ANC missing for 26 cases and 6 controls

**Information on number of ultrasounds missing for 7 cases and 35 controls

***Information on the ‘use of IPT’ and ‘number of IPTs taken’ missing for 7 cases and 9 controls

^F^ Fisher Exact test

### Medical determinants of stillbirth

Medical factors that were significantly associated with stillbirth in the bivariate analysis included syphilis (OR: 5.0; 95% CI: 1.003–39.15, *p* = 0.043), HIV (OR: 3.32; 95% CI: 1.44-8.00, *p* = 0.003), hypertensive disorder in pregnancy (OR: 5.99; 95% CI: 2.64–13.58, *p* < 0.001), malaria (OR: 4.98; 95% CI: 2.16–11.47, *p* < 0.001), and alcohol consumption (OR: 3.75; 95% CI: 1.58–8.89, *p* = 0.002) (Table 4).

**Table 4:** Medical factors associated with stillbirths

Variable	Category	Cases n (%)	Controls n (%)	OR [95% CI]	p value
Anaemia	Yes	27 (27.0)	37 (18.5)	1.63 [0.92–2.88]	0.090
	No	73 (73.0)	163 (81.5)	1	
Syphilis	Yes	5 (5.0)	2 (1.0)	5.00 [1.003–39.15]	**0.043**
	No	95 (95.0)	198 (99.0)	1	
HIV	Yes	15 (15.0)	10 (5.0)	3.32 [1.44-8.00]	**0.003**
	No	85 (85.0)	190 (95.0)	1	
Hypertensive Disorder	Yes	22 (22.0)	9 (4.5)	5.99 [2.64–13.58]	**< 0.001**
	No	78 (78.0)	191 (95.5)	1	
Malaria	Yes	19 (19.0)	9 (4.5)	4.98 [2.16–11.47]	**< 0.001**
	No	81 (81.0)	191 (95.5)	1	
Fibroid in pregnancy	Yes	2 (2.0)	1 (0.5)	4.06 [0.36–45.34]	0.256
	No	98 (98.0)	199 (99.5)	1	
Smoking	Yes	2 (2.0)	1 (0.5)	4.06[0.36–45.33]	0.259^F^
	No	98 (98.0)	199 (99.5)	1	
Alcohol	Yes	15 (15.0)	9 (4.5)	3.75 [1.58–8.89]	**0.002**
	No	85 (85.0)	191 (95.5)	1	

CI: Confidence Interval; OR: Odds ratio; HIV: Human Immuno-deficiency Virus ^F^ Fisher Exact test

### Foetal determinants of stillbirth

Foetal factors that were associated with stillbirth in the bivariate analysis included birth weight < 2.5 kg (OR: 13; 95% CI: 6.78–24.99; *p* = 0.000), congenital malformations (OR: 8.61; 95% CI: 1.79–41.34; *p* = 0.003), gestational age < 37 completed weeks (OR: 8.28; 95% CI: 4.50-15.23; *p* < 0.0001), and cord prolapse (OR: 6.32; 95% CI: 1.25–31.9; *p* = 0.018) (Table 5).

**Table 5:** Foetal factors associated with stillbirth

Variable	Category	Cases n (%)	Controls n (%)	OR [95%CI]	p value
Weight of Baby (grams)	<2500 g	55 (55.0)	16 (8.0)	13.01[6.78–24.99]	**< 0.001**
	2500–3999 g	42 (42)	159 (79.5)	1	
	≥4000	3 (3.0)	25 (12.5)	0.45 [0.13–1.58]	0.309
Sex of the baby	Male	53 (53.0)	107 (53.5)	1	
	Female	47 (47.0)	93 (46.5)	1.02 [0.63–1.65]	0.935
Congenital malformations	Yes	8 (8.0)	2 (1.0)	8.61 [1.79–41.34]	**0.003**
	No	92 (92.0)	198 (99.0)	1	
Term	< 37 weeks	48 (48.0)	21 (10.5)	8.28 [4.50-15.23]	**< 0.001**
	37–41 weeks	51 (51.0)	176 (88.0)	1	
	≥ 42 weeks	1 (1.0)	3 (1.5)	1.58 [0.61–4.09]	0.429
Number of foetuses	singleton	94(94.0)	190(95.0)	1	
	multiple	6(6.0)	10(5.0)	1.21[0.43–3.44]	0.717
Cord prolapse	Yes	6(6.0)	2(1.0)	6.32[1.25–31.90]	**0.018**
	No	94(94.0)	198(99.0)	1	

CI: Confidence Interval; OR: Odds ratio

### Factors associated with stillbirth after adjusting for confounders (multivariable logistic regression)

Factors that remained associated with stillbirth after multivariable logistic regression were: nulliparity (aOR = 3.89; 95% CI: 1.19–12.71; *p* = 0.025), not attending ANC (aOR = 104; 95% CI: 3.17–3472; *p* = 0.009), history of stillbirth (aOR = 44; 95% CI: 7-270; *p* < 0.0001), gestational age at PROM (aOR = 11.32; 95% CI: 1.44–42.54; *p* < 0.021), placenta abruption (aOR = 14; 95% CI: 2.4–84; *p* = 0.003), hypertensive disorder in pregnancy (aOR = 18; 95% CI: 3.4–98; *p* = 0.001), malaria (aOR = 8; 95% CI: 1.51-42; *p* = 0.015), alcohol consumption (aOR = 9; 95% CI: 1.72-50; *p* = 0.01), birth weight less than 2500 g (aOR = 16; 95% CI: 3.0–89; *p* = 0.001), and congenital malformations (aOR = 12.6; 95% CI: 1.06–149.7; *p* = 0.045) (Table 6).

**Table 6:** Factors associated with stillbirth (multivariable analysis)

Variables		aOR [95% CI]	p value
Level of education	Basic	Reference	
	Secondary	0.46 [0.12–1.76]	0.254
	University	0.82 [0.23–2.94]	0.761
	No formal	1.16 [0.07–18.28]	0.915
Marital status	Single	1.79 [0.63–5.15]	0.277
	Married	Reference	
Parity	0	3.89 [1.19–12.71]	**0.025**
	1–3	Reference	
	≥ 4	0.43 [0.04–4.65]	0.491
ANC location	No ANC	104.91 [3.17–347]	**0.009**
	Health centre	2.01 [0.54–7.51]	0.298
	District H	2.19 [0.49–9.79]	0.303
	Regional H	Reference	
Number of ANC visits	0	0.77 [0.26–2.32]	0.645
	1–2	0.09 [0.01–0.60]	**0.013**
	3–4	0.77 [0.26–2.32]	0.645
	≥ 5	Reference	
History of stillbirth	Yes	43.59 [7.02-270.63]	**< 0.001**
	No	Reference	
Number of ultrasound scans	0	3.82 [0.51–28.75]	0.192
	1–2	1.41 [0.21–9.56]	0.726
	≥ 3	Reference	
TORCH	Done	0.53 [0.17–1.63]	0.269
	Not done	Reference	
Infections in pregnancy	Yes	2.74 [0.48–15.55]	0.256
	No	Reference	
Abruptio placenta	Yes	14.14 [2.39–83.77]	**0.003**
	No	Reference	
Syphilis	Yes	5.98 [0.28-128.44]	0.256
	No	Reference	
HIV	Yes	0.76 [0.07–8.71]	0.827
	No	Reference	
Hypertensive disorder in pregnancy	Yes	18.24 [3.39–98.23]	**0.001**
	No	Reference	
Malaria	Yes	8.01 [1.51–42.45]	**0.015**
	No	Reference	
Alcohol	Yes	9.34 [1.72–50.56]	**0.010**
	No	Reference	
Birth weight group	< 2500 g	16.31 [3.00-88.75]	**0.001**
	2500–3999 g	Reference	
	≥ 4000 g	0.78 [0.07–8.20]	0.838
Congenital malformation	Yes	12.61 [1.06-149.68]	**0.045**
	No	Reference	
Term	< 37	1.08 [0.20–5.70]	0.927
	37–41	Reference	
	≥ 42	1.73 [0.29–10.27]	0.548
Cord prolapse	Yes	0.41 [0.02–6.80]	0.530
	No	Reference	
PROM	Yes	1.45 [0.15–14.52]	0.751
	No	Reference	
GA at PROM (weeks)	**28 to < 37**	11.32 [1.44–42.54]	**0.021**
	≥ 37	Reference	
Oligohydramnios	Yes	7.56 [1.66–21.78]	0.069
	No	Reference	
Anaemia	Yes	1.04 [0.45–2.40]	0.972
	No	Reference	

CI: Confidence Interval; aOR: adjusted Odds ratio; ANC: antenatal care; TORCH: Toxoplasmosis, rubella, cytomegalo virus, herpes; HIV: Human Immuno-deficiency Virus

## Discussion

### Summary of key findings

This study aimed to determine the stillbirth rate and factors associated with stillbirth at the Bamenda Regional Hospital, North West region, Cameroon. A stillbirth rate of 8.9 per 1000 live births was obtained. After multivariable logistic regression, nulliparity (parity 0), a past history of stillbirth, premature rupture of membranes at less than 37 weeks of gestation, no visits, placental abruption, hypertensive disorder in pregnancy, malaria, birth weight < 2500 g, and congenital malformations remained positively associated with stillbirth.

### Rate of stillbirth

We found a stillbirth rate of less than 10 stillbirths per thousand live births (8.9‰, 95% CI: 7.4–10.7), which was much lower than the reported values in previous studies in Cameroon, which were 26‰ in a similar study conducted in the Buea Regional Hospital in 2017 [13] and 33.7 and 36.5 in the Buea Regional Hospital and Limbe Regional Hospital, respectively, in 2020 [8]. Our rate was also lower than reported rates in other countries, such as 46.9‰ in Nigeria in 2015 [12] and 16‰ in India in 2017 [14]. Although regional differences in health care could account for varying rates, the study periods of these studies were many years back; thus, our reduced rate could imply improvement in care over time. However, our stillbirth rate was slightly higher than the 6.2‰ reported in Latvia in 2019 [15]. The latter study, although its cut-off for stillbirth was taken at 22 weeks, was a cohort study with close monitoring of pregnant women and a probable higher level of care received by the pregnant women.

### Factors associated with stillbirth

An association between stillbirth and nulliparity was found in this study. Similar results have been reported in earlier studies in Pakistan by Nazli et al. in 2009 [16] and in Nepal by Khadka et al. in 2022 [17]. Studies in India by Avachat et al. in 2015 [18] and in Burkina Faso by Millogo et al. in 2016 [19] had contradictory results, showing no significant association between parity and the risk of stillbirths, while another study in India by Shyam et al. in 2016 [20] showed that high parity was associated with increased risk. Women with higher parity turned to stay away from ANC while counting on their previous pregnancy experience. Higher parity was found to be associated with reduced ANC visits in a study in Rwanda by Miller et al. in 2021 [21]. However, a systematic review on factors associated with stillbirth in LMICs by Aminu et al. in 2014 [22] concluded that women who had never delivered were at higher risk of stillbirth, which corroborates with the findings of this study. Although all pregnant women ought to receive focalised ANC, this finding beckons that more emphasis should be made in the follow-up of nulliparous pregnant women.

Having a past history of stillbirth was found to have higher odds of having a stillbirth by over 40-fold. This was consistent with studies performed in India by Sutapa et al. in 2016 [23], in Ghana by Yatich et al. in 2010 [24] and in Nigeria by Friday et al. in 2019 [25], which revealed a substantially increased risk of stillbirth with a previous history of stillbirth. The likely explanation for this association is possibly the presence of a triggering factor that is to be screened or investigated. In addition, more credit to the importance of antenatal care should be given since most of these conditions could be managed effectively and prevented when women attend antenatal care regularly [25].

The odds of stillbirth was14-fold higher among those with placental abruption in our study. This finding is consistent with many other studies, such as in Nigeria, Pakistan and Tanzania [12, 16, 26]. In abruption, the placenta separates from the wall of the uterus before birth, which can lead to reduced oxygen and nutrient supply to the foetus, and in some cases, it might be concealed or the remaining placental surface that has not detached is too small to sustain the foetus. Hypertensive disorder is a known major risk factor for abruptio placenta, and our study showed a significant association between hypertensive disorder in pregnancy and stillbirth. This finding corroborates with other findings obtained in Northern Tanzania by Chuwa et al. in 2017 [27] and in Nepal by Khadka et al. in 2022 [17]. In addition to abrupt changes, hypertension can lead to stillbirth by causing chronic placental insufficiency with chronic foetal distress, resulting in intra-uterine growth restriction (IUGR) and eventual death [28]. This makes it very imperative that women with hypertensive disorders should be closely monitored and timely interventions should be done to reduce foetal compromise.

This study showed that the odds of stillbirth was higher amongst those who had malaria in pregnancy. Other studies have also shown higher odds of stillbirth with malaria, such as in the studies by Yatich et al. in Ghana [24] and by Aminu et al. in a systematic literature review of factors associated with stillbirth in LMICs [22]. Malaria is endemic in many African countries, including Cameroon, where several strategies have been put in place to combat malaria, especially among pregnant women. It is therefore important to emphasise on malaria prevention strategies among pregnant women in order to prevent malaria in pregnancy which could lead to stillbirth.

This study demonstrated a significant positive association between stillbirth and alcohol consumption, although it did not differentiate in the volumes of alcohol consumed. This finding were similar to those obtained by Chuwa et al. in Tanzania 2017 [27] and Geelhoed et al. in Mozambique in 2015 [29]. Although a threshold for alcohol consumption was not made, it could be safer avoiding alcohol during pregnancy.

As seen in this study, the odds of stillbirth was 16-fold higher amongst low-birth-weight babies. Other studies have reported increased odds of stillbirth among low-birth-weight infants [8, 10, 17, 18]. It has been suggested that low-birth-weight infants could be less adapted to withstand labour and the transition to life outside of the uterus [8]. The odds of having a stillbirth is 12-fold higher amongst babies with congenital malformations. A study performed in Cameroon by Charlotte et al. reported similar findings in 2015 [30]. Other studies from Cameroon have also reported an association between congenital malformations and stillbirth [20, 31, 32]. Therefore, more emphasis must be placed on prenatal counselling and early screening for antenatal infections to reduce the risk of preventable congenital malformations, thus reducing the risk of stillbirth.

### Limitations and strengths of our study

#### Limitations

This study was a retrospective study, and like most retrospective studies, missing data and reported bias are difficult to eliminate. Additionally, our study, being a hospital-based study, may impose a selection bias since only those who came to the hospital were included. More importantly, the factors identified are just associations and do not necessarily imply a causal association. Although some exposure variables such as fibroid in pregnancy, smoking, and cord prolapse were not significantly associated with the outcome, it is thought that, this study was not powered enough to detect associations as the number of exposed cases and controls were small.

### Strengths

This study was carried out in the main referral hospital of the North West region, which receives more pregnant women and conducts more deliveries than any other facility. Additionally, its relatively low-cost services and availability of permanent obstetricians makes it open to receive women from all social classes, hence making our results generalizable to the entire northwest region of Cameroon.

## Conclusion

The stillbirth rate in the BRH is 8.9 per 1000 live births, which is lower than the rates in other areas in Cameroon and the target set by the WHO’s Every New-Born Action Plan in all countries by 2030. Risk factors for stillbirth include nulliparity, not attending ANC, history of stillbirth, placental abruption, hypertensive disorder in pregnancy, malaria, alcohol consumption, birth weight less than 2500 g, and congenital malformations. Close ANC follow-up of women with such associated factors is recommended.

## Data Availability

The dataset used in this study is available from the corresponding author upon reasonable request.

## Abbreviations

aOR: Adjusted odds ratio
ANC: Antenatal care
BRH: Bamenda Regional Hospital
CI: Confidence interval
IPT: Intermittent preventive treatment
IUGR: Intrauterine Growth Restriction
LMIC: Low- and middle-income country
OR: Odds ratio
TORCH: Toxoplasmosis, rubella, cytomegalovirus, herpes simplex
WHO: World Health Organization

## References

[1] World Health Organization (2016). Making every baby Count: audit and review of Stillbirths and neonatal deaths.

[2] Harrison JE, Weber S, Jakob R, Chute CG. ICD-11: an international classification of diseases for the twenty-first century. BMC Med Inform Decis Mak. 2021;21(Suppl 6):206. 10.1186/s12911-021-01534-6. 10.1186/s12911-021-01534-6 PMC8577172 34753471

[3] Tesema GA, Tessema ZT, Tamirat KS, Teshale AB (2021). Prevalence of stillbirth and its associated factors in East Africa: generalized linear mixed modelling. BMC Pregnancy Childbirth.

[4] Hug L, You D, Blencowe H, Mishra A, Wang Z, Fix MJ (2021). Global, regional, and national estimates and trends in stillbirths from 2000 to 2019: a systematic assessment. Lancet.

[5] Blencowe H, Cousens S, Jassir FB, Say L, Chou D, Mathers C (2016). National, regional, and worldwide estimates of stillbirth rates in 2015, with trends from 2000: a systematic analysis. Lancet Glob Health.

[6] Anyichie NE, Nwagu EN (2019). Prevalence and maternal sociodemographic factors associated with stillbirth in health facilities in Anambra, South–East Nigeria. Afr Health Sci.

[7] Amani A, Nansseu JR, Ndeffo GF, Njoh AA, Cheuyem FZL, Libite PR (2022). Stillbirths in Cameroon: an analysis of the 1998–2011 demographic and health surveys. BMC Pregnancy Childbirth.

[8] Egbe TO, Ewane EN, Tendongfor N (2020). Stillbirth rates and associated risk factors at the Buea and Limbe regional hospitals, Cameroon: a case–control study. BMC Pregnancy Childbirth.

[9] Murphy S, Cacciatore J (2017). The psychological, social, and economic impact of stillbirth on families. Semin Fetal Neonatal Med.

[10] Mohammed-Ahmed A, Abdullahi A, Beshir F (2022). Magnitude and associated factors of stillbirth among women who gave birth at Hiwot Fana Specialized University Hospital, Harar, eastern Ethiopia. Eur J Midwifery.

[11] McClure EM, Goldenberg RL (2009). Stillbirth in developing countries: a review of causes, risk factors and prevention strategies. J Matern-Fetal Neonatal Med.

[12] Suleiman BM, Ibrahim HM, Abdulkarim N (2015). Determinants of Stillbirths in Katsina, Nigeria: A Hospital-based study. Pediatr Rep.

[13] Anu NB, Nkfusai CN, Evelle MNM, Efande LE, Bede F, Shirinde J (2019). Prevalence of stillbirth at the Buea Regional Hospital, Fako Division south–west region, Cameroon. Pan Afr Med J.

[14] Newtonraj A, Kaur M, Gupta M, Kumar R (2017). Level, causes, and risk factors for stillbirth: a population-based case control study from Chandigarh, India. BMC Pregnancy Childbirth.

[15] Zile I, Ebela I, Rumba-Rozenfelde I (2019). Maternal risk factors for Stillbirth: A Registry–Based Study. Med (Mex).

[16] Hossain N, Khan N, Khan NH (2009). Obstetric causes of stillbirth at low socioeconomic settings. J Pak Med Assoc.

[17] Khadka D, Dhakal KB, Dhakal A, Rai SD (2022). Stillbirths among pregnant women admitted to the department of obstetrics and gynaecology in a tertiary care centre: a descriptive cross-sectional study. J Nepal Med Assoc.

[18] Avachat SS, Phalke DB, Phalke VD (2015). Risk factors associated with stillbirths in the rural area of Western Maharashtra, India. Arch Med Health Sci.

[19] Millogo T, Ouédraogo GH, Baguiya A, Meda IB, Kouanda S, Sondo B (2016). Factors associated with fresh stillbirths: a hospital-based, matched, case–control study in Burkina Faso. Int J Gynecol Obstet.

[20] Shyam P (2016). Analysis of risk factors for stillbirth: a hospital-based study in a tertiary care centre. Int J Reprod Contracept Obstet Gynecol.

[21] Miller P, Afulani PA, Musange S, Sayingoza F, Walker D (2021). Person-centered antenatal care and associated factors in Rwanda: a secondary analysis of program data. BMC Pregnancy Childbirth.

[22] Aminu M, Unkels R, Mdegela M, Utz B, Adaji S, van den Broek N (2014). Causes of and factors associated with stillbirth in low- and middle-income countries: a systematic literature review. BJOG.

[23] Neogi SB, Negandhi P, Chopra S, Das AM, Zodpey S, Gupta RK (2016). Risk factors for Stillbirth: findings from a Population-based case–control study, Haryana, India. Paediatr Perinat Epidemiol.

[24] Jolly PE, Yatich NJ, Funkhouser E, Ehiri JE, Agbenyega T, Stiles JK et al. Malaria, intestinal helminths and other risk factors for stillbirth in Ghana. Infect Dis Obstet Gynecol. 2010; 2010:350763. 10.1155/2010/350763. 10.1155/2010/350763 PMC2850132 20379355

[25] Okonofua FE, Ntoimo LFC, Ogu R, Galadanci H, Mohammed G, Adetoye D et al. Prevalence and determinants of stillbirth in Nigerian referral hospitals: a multicentre study. BMC Pregnancy Childbirth. 2019;19(1). 10.1186/s12884-019-2682-z. 10.1186/s12884-019-2682-z PMC6937841 31888536

[26] Kidanto H, Msemo G, Mmbando D, Rusibamayila N, Ersdal H, Perlman J (2015). Predisposing factors associated with stillbirth in Tanzania. Int J Gynecol Obstet.

[27] Chuwa FS, Mwanamsangu AH, Brown BG, Msuya SE, Senkoro EE, Mnali OP (2017). Maternal and fetal risk factors for stillbirth in Northern Tanzania: a registry-based retrospective cohort study. PLoS ONE.

[28] Low JA, Boston RW, Cervenko FW (1970). A clinical classification of the mechanisms of Perinatal Wastage. Can Med Assoc J.

[29] Geelhoed D, Stokx J, Mariano X, Mosse Lázaro C, Roelens K (2015). Risk factors for stillbirths in Tete, Mozambique. Int J Gynaecol Obstet.

[30] Tchente NC, Nzesseu DA, Brulet C, Barla E, Belley-Priso E (2015). Prenatal Diagnosis of Congenital Malformations in Douala General Hospital. Open J Obstet Gynecol.

[31] Kebede E, Kekulawala M. Risk factors for stillbirth and early neonatal death: a case–control study in tertiary hospitals in Addis Ababa, Ethiopia. BMC Pregnancy Childbirth. 2021;21(1):641. 10.1186/s12884-021-04025-8. 10.1186/s12884-021-04025-8 PMC8456546 34548064

[32] Tolefac PN, Tamambang RF, Yeika E, Mbwagbaw LT, Egbe TO (2017). Ten years analysis of stillbirth in a tertiary hospital in Sub-sahara Africa: a case control study. BMC Res Notes.

